# A Survey of the Knowledge and Beliefs of Retired Men about Prostate Cancer Screening Based on Health Belief Model

**Published:** 2014-10

**Authors:** Fariba Ghodsbin, Maryam Zare, Iran Jahanbin, Ali Ariafar, Sareh Keshavarzi

**Affiliations:** 1 Community Based Psychiatric Care Research Center, Department of Community Health Nursing, School of Nursing and Midwifery, Shiraz University of Medical Sciences, Shiraz, Iran;; 2 Department of Community Health Nursing, School of Nursing and Midwifery, Shiraz University of Medical Sciences, Shiraz, Iran;; 3 Urology Oncology Research Center, Shiraz University of Medical Sciences, Shiraz, Iran;; 4 Department of Epidemiology, School of Health, Shiraz University of Medical Sciences, Shiraz, Iran

**Keywords:** Prostatic Neoplasms, Early Detection of Cancer, Knowledge, Retirement

## Abstract

**Background: **Prostate cancer has been reported as the second leading cause of cancer death and the most common cancer diagnosed in men. Since Health Belief Model (HBM) has been intensively used for assessing health beliefs associated with cancer screening behaviors, we aimed to investigate the level of knowledge and health beliefs about prostate cancer screening among retired men.

**Methods: **In this descriptive study carried out in 2013, we enrolled 180 men aged 50-70 years who were retired from Shiraz Education Department. Data were collected using three questionnaires including demographic questionnaire, HBM and the Knowledge about Prostate Cancer Screening questionnaires by interviewing the participants.

**Results: **Our findings showed that 95.6% and 85.6% of the interviewees had no experience of digital rectal examination (DRE) and prostatic-specific-antigen (PSA) testing for prostate cancer screening, respectively. 86.1% of men had no knowledge about such screening. 12.7% of the respondents reported good knowledge scores. 74.4% and 90.5% of them had good health motivation and perceived benefits scores, respectively. 81.6% of them revealed intermediate scores for perceived barriers. Moreover, 32.7% and 7.2% of the subjects reported good severity and susceptibility scores, respectively.

**Conclusion: **Developing an assessment based on HBM could be effective in designing and implementing educational programs by helping to identify the needs and priorities of the target population.

## Introduction

Prostate cancer is the most commonly diagnosed cancer and the second leading cause of cancer death among men in the United States. According to the official census published by American cancer society in 2013, one in six American men is prone to develop prostate cancer during his lifetime.^1^

According to the published statistics in Iran, its age-standardized incidence rate during 2003 to 2008 was 4.69, 7.16, 14.04, 16.65, and 16.02 per 100,000 men, respectively, indicating an increasing trend of the disease during the above mentioned years.^2^

Besides, almost 60% of all prostate cancer cases are diagnosed in men aged 65 years and older and 97% of cases occur in men aged 50 and older .The American Cancer Society suggests that beginning at age 50, the men who are at average risk of prostate cancer, with at least 10 years of life expectancy, need to be screened for early prostate cancer detection. Moreover, they should receive sufficient information about the potential benefits of screening so that they can make an informed decision about testing based on their personal values and preferences. Those who are at higher risk of developing such disease should also have consultations with their health care provider beginning at age 45 and 40.^1^

Furthermore, two European studies revealed that men who received prostate-specific antigen (PSA) screening tests were at lower risk of death from prostate cancer. According to the mentioned studies, increasing age, African ancestry, and a family history were the only risk factors of such disease.^1^

Research has also demonstrated that regular screening with digital rectal exam (DRE) and PSA testing help diagnose such cancer at early stages. Similarly, Tingen, et al. (1998) pointed out that 9 out of 10 men could survive a minimum of five years with early detection through screening; however, with late diagnosis, only 3 out of 10 could have a five year survival rate.^3^

It is supposed that knowledge about the disease is an effective factor in men’s participation in screening programs since the studies have shown that men with higher levels of knowledge show higher tendency towards the screening.^4^Counseling and education, especially if done based on a specific protocol, can possibly lead to behavioral changes. Therefore, different educational models such as health belief and planned behavior models as well as social cognitive theory have been evaluated to explain such changes.^5^

Health Belief Model (HBM) has been intensively used for assessing health beliefs associated with cancer screening behaviors. HBM is a cognitive model attempting to identify and predict health behaviors.^6^ To perform healthy behaviors, according to such model, one should initially perceive the risk of contracting the health condition of concern (perceived susceptibility) and the seriousness and severity of the consequences and complications derived from such condition with its all physical, psychological, social and economic dimensions (perceived severity) by receiving positive cues in the form of incentives from external or internal environments (cues to action) and finally take action after believing in its suitability and applicability.^7^

Also, external environment for individuals includes physician, nurse and health care workers.^8^ When an individual perceives the factors that inhibit the action (perceived barrier) and find them more costly than the benefits of the action (perceived benefit), he/she decides to undergo screening.^9^ Hence, we used HBM which has more emphasis on prevention as a reference framework.

Perceived susceptibility, severity, benefits and barriers as well as health motivations are all the constructs of this model which are investigated in our study. We aimed to examine the level of knowledge and health beliefs about prostate cancer screening among retired men. 

## Materials and Methods

This study was approved by the Ethics Committee of Shiraz University of Medical Sciences (Ethics Committee Approval Number: ct-92-6721). The sample size was calculated as 184 based on the data of similar studies and using Power SSC statistical software (power: 80%, α: 0.05, mean difference: 2.36, SD: 12.1, and error=%5). In this descriptive study, we enrolled 180 men aged 50-70 years during April to October 2013.4 incomplete copies of the questionnaire were removed. We selected our participants from the population of men who were retired from Shiraz Education Department, using a simple random sampling method. The researchers referred to the list of the males retired from Shiraz Education Department, using table of random numbers.

Inclusion criteria were willingness to participate in the study, completing the written informed consent, no history of prostate cancer, benign prostatic hyperplasia with obvious clinical symptoms, as well as age of 50 to70 years. The only exclusion criterion was unwillingness to participate in the study.

After explaining the aims of the study, written informed consent was obtained from all the participants and their anonymity and confidentiality were guaranteed. Data were collected by the researcher and a trained research assistant after interviewing the participants using three questionnaires including demographic, HBM and the Knowledge about Prostate Cancer Screening questionnaires. 

Demographic questionnaire which was developed by the researcher comprised 13 items on demographic characteristics of interviewees(including age, marital status, educational level and monthly income), history of undergoing prostate cancer screening using DRE and PSA testing, a family history of such cancer, knowledge about the disease as well as the methods of acquiring knowledge about it.

The Knowledge about Prostate Cancer Screening Questionnaire was developed by Weinrich and colleagues (2004) to measure the level of knowledge about prostate cancer and its screening. It consisted of 12 questions to be answered with the options of “true”, “false” and “I don’t know”. “I don’t know” responses were scored as incorrect. The scores ranged from 0 to 12 with higher scores reflecting higher level of knowledge. Scores lower than 7, 7-9 and 10-12 were considered as low, intermediate and good, respectively.^10^

HBM questionnaire for prostate cancer screening which was designed by Capık and Gözüm (2011) included 41 items in a 5-point Likert scale anchored at 1=completely disagree and 5=completely agree. The scale consisted of 41 questions and 5 sub-scales including perceived susceptibility (5 items), perceived severity (5 items), health motivations (10 items), perceived barriers (15 items), and perceived benefits (7 items).^11^ The scores between maximum and minimum scores were classified into three levels of low, intermediate and good, respectively.

To ensure about the reliability of both questionnaires, they were translated into Persian and back-translated by the researcher. After performing a pilot study on 30 retired clerks, Kuder Richardson 20 coefficient was calculated as 0.98 for the Knowledge about Prostate Cancer Screening Questionnaire. Cronbach’s alpha was also calculated as 0.83 for HBM questionnaire. Moreover, the validity of the two questionnaires was determined by content validity method, using the opinions of 10 urologists and professors of nursing faculty. Statistical analysis was done using SPSS, version 19. The quantitative data are demonstrated as a mean and standard deviation and the qualitative data are shown in the tables. 

## Results

The age range of the participants was 50-70 years and their mean±SD age was 57.4±5.06. The rate of the respondents who had no family history of prostate cancer and no experience of performing DRE and PSA testing for screening was reported as 87.2%, 95.6% and 85.6%, respectively.86.1% of the males had no knowledge about prostate cancer screening; however, the other participants knew about it and reported television (48%), magazines and newspapers (20%), a family member with such disease (12%), radio (8%), physicians (8%), and friends (4%) as their source of knowledge. Table 1 shows demographic characteristics of all the participants.

**Table 1:** Frequency distribution of participants’ personal characteristics (N=180)

**Personal Characteristics**		**Frequency (N)**	**Percentage (%)**
Educational Level	Undergraduate (degrees lower than high school diploma)	10	5.6
	High school Diploma	41	22.8
	Associate’s degree	54	30
	Bachelor’s degree	62	34.4
	Master’s degree and Postgraduate Diploma/Certificate	13	7.2
Monthly income (Rials)	4.000.000-6.000.000	32	17.7
	60.000.000-10.000.000	125	69.5
	>10.000.000	25	12.8
Marital Status	Single	6	3.3
	Married	171	95
	Divorced	1	0.6
	Widower	2	1.1

Table 2 shows the mean and standard deviation of the Health Belief Model component and knowledge of all the participants and the scores obtained from knowledge and HBM component were shown at three levels of good, intermediate and low.

**Table 2:** Distribution of the mean scores (±SD) of perceived susceptibility, benefits, severity and barriers as well as health motivation and knowledge in the participants (N=180)

**Variable**	**Mean±SD**	**Levels of Variables**					
		**Low**		**Intermediate**		**Good**	
		**Frequency** **(N)**	**Percentage** **(%)**	**Frequency** **(N)**	**Percentage** **(%)**	**Frequency** **(N)**	**Percentage** **(%)**
Perceived Susceptibility	14.11±4.05	58	32.2	109	60.5	13	7.2
Perceived Severity	12.88±3.43	32	17.7	89	49.4	59	32.7
Perceived Barriers	45.60±7.42	20	11.1	147	81.6	13	7.2
Perceived Benefits	29.75±3.61	2	1.1	15	8.3	163	90.5
Health motivation	39.64±6.41	4	2.2	42	23.3	134	74.4
Knowledge	7.76±1.71	39	21.6	118	65.5	23	12.7

87.3% of the respondents reported low and intermediate knowledge scores. 74.4% and 90.5% of them had good health motivation and perceived benefits scores, respectively.92.8% of them reported high and intermediate scores for perceived barriers. Moreover, 67.3% and 92.8% of them reported low and intermediate severity and susceptibility scores, respectively (table 2).

## Discussion

76% of the participants mentioned mass media such as television, radio and journals as their source of obtaining knowledge about prostate cancer screening. As to this point, Askeri Nejad and Bakhshi (2000) urged to use mass media for improving the participants’ knowledge.^12^ Therefore, using HBM, which is beneficial for designing educational programs, changing attitudes and especially improving knowledge, helps to make more people aware of prostate cancer screening programs.

The percentage of our participants who had an experience of performing DRE and PSA testing for prostate cancer screening was reported as 4.4% and 14.4%, respectively, indicating more acceptability of PSA testing compared with DRE among men. The studies conducted by Nagler et al. (2005) and Oliver et al. (2011) also confirmed our findings.^13^^,^^14^

The results revealed that only 12.7% of the interviewees reported a good level of knowledge. Our finding was consistent with those of Gozum and Capik (2010) who studied the validation of HBM scale for prostate cancer screenings in Turkey and reported a low level of knowledge among Turkish men in such field.^15^Similarly, in a study done by Ford et al (2006), African-American men revealed poor level of knowledge regarding prostate cancer screening.^16^

Rezaeian et al (2007) investigated the knowledge, attitude and practice of pensioners towards prevention of prostate cancer. However, they found out that 55% of the respondents demonstrated a good level of knowledge; they believed that such rate was not acceptable. Moreover, they necessitated developing programs for improving men’s knowledge about early diagnosis and treatment of prostate cancer as the disease is treatable at early stages.^4^

Our results also showed that 74.4% and 90.5% of men reported good level of health motivations and perceived benefits, respectively. According to our result, our participants believed that taking effective preventive actions such as prostate cancer screening behaviors could result in early diagnosis of the disease. Such finding was consistent with those of Mahmoodiand Ramazani (2011) and Namdar et al. (2012); however, our result is in contrast with that of Capık and Gözüm (2012) who studied the validation of HBM scale for prostate cancer screenings in Turkey and reported mean scores of 3.33 and 6.24 for health motivations and perceived benefits before intervention, respectively among Turkish men in such field. Our results (64.39 and 75.29) show that Turkish men have lower levels of motivation and perceived benefits.^6^^,^^17^^,^^18^

Our results indicated that only 7.2% of the respondents reported a low level of perceived barriers (lower mean scores of perceived barriers reflects a more satisfactory results). Also, it was shown that although our participants believed prostate cancer screening could be useful for early diagnosis of the disease, they believed that there were many barriers for doing so. This finding was consistent with the studies of Namdar et al. (2012) on cervical cancer and Taghdisi et al. (2011) who examined the effect of HBM on prevention of urinary infections in pregnant women.^17^^,^^19^

Performing educational interventions to raise the awareness and improve knowledge about prostate cancer screening seem necessary due to some reasons. On one hand, according to several studies conducted by researchers in this field, lack of knowledge has been considered as the most important perceived barrier. On the other hand, perceived barriers are directly associated with early diagnosis of the disease and participation in the screening programs.^15^^,^^16^^,^^20^^,^^21^

Moreover, such subscale is the most powerful dimensions of HBM in predicting people’s behavior and based on such model, individuals decide to participate in screening programs only when they find the inhibiting factors (perceived barriers) more costly than its benefits (perceived benefits).^9^

We also found that the mean scores of perceived susceptibility and severity were low as only 32.7% and 7.2% of the participants reported good level of perceived susceptibility and severity. Despite the high incidence rate of prostate cancer, such finding reflects the fact that the studied men considered themselves at low risk of such disease and disregarded its severity and complications. Our finding was similar to that of Sajadi Hazaveh and Shamsi (2011) who evaluated the mothers’ behavior about prevention of febrile seizure in children and that of Daniel and Messer (2002) who conducted a similar study on the patients with type 2 diabetes mellitus.^22^^,^^23^

Therefore, it seems necessary to consider the perceived susceptibility, severity and barriers as the focus areas of further educational intervention to create sensitivity and remove barriers. We expect to make men more sensitive to the risk of prostate cancer by implementing educational programs and public notifications.

Considering the fact that the present study is a clinical trial study, the small sample size and selecting samples only from the limited population of retired males of Shiraz Education Department were the limitations of our study. Thus, large scale studies with larger sample size selected randomly from all parts of the society are recommended to obtain more generalizable results for macro-planning in the health sector.

This research was a primary clinical trial study with the aim of finding out the status of these participants so that future educational programs could be designed according to clients’ needs. Selecting samples only from the limited population of retired males of Shiraz Education Department was the limitation of our study. Thus, performing such studies on general population is recommended to obtain more generalizable results for macro-planning in the health sector. 

## Conclusion

Developing an assessment based on HBM could be effective in designing and implementing educational programs by helping to identify the needs and priorities of the target population. Educational programs should be designed so that the weaknesses of the clients are identified and eliminated based on their needs; in this way, we will be able to help individuals gain more knowledge concerning their health. 
